# Increased CDC6 Expression Associates With Poor Prognosis in Patients With Clear Cell Renal Cell Carcinoma

**DOI:** 10.3389/fonc.2021.666418

**Published:** 2021-05-24

**Authors:** Yicong Yao, Yi Wang, Denglong Wu, Baoying Hu

**Affiliations:** ^1^ Department of Urology, Tongji Hospital, School of Medicine, Tongji University, Shanghai, China; ^2^ School of Medicine, Tongji University, Shanghai, China; ^3^ Department of Urology, Affiliated Hospital of Nantong University, Nantong, China; ^4^ Department of Immunology, Medical College, Nantong University, Nantong, China

**Keywords:** CDC6, prognosis, clear cell renal cell carcinoma, survival, pathway

## Abstract

**Background:**

CDC6 (Cell division control protein 6), located at chromosome 17q21.3, plays an important role in the early stage of DNA replication and has unique functions in various malignant tumors. Here, we evaluate the relationship between CDC6 expression and oncology outcomes in patients with clear cell renal cell carcinoma (ccRCC).

**Methods:**

A retrospective analysis of 118 ccRCC patients in Affiliated Hospital of Nantong University from 2015 to 2017 was performed. Triplicate tissue microarrays (TMA) were prepared from formalin-fixed and paraffin-embedded specimens. Immunohistochemistry (IHC) was conducted to evaluate the relationship between CDC6 expression and standard pathological features and prognosis. The RNA sequencing data and corresponding clinical information were acquired from the TCGA database. GSEA was used to identify signal pathways related to CDC6. Cox regression analysis was used to assess independent prognostic factors. In addition, the relationship between CDC6 and immunity was also investigated.

**Results:**

The results of Kaplan–Meier curve indicated that the OS of the patients with high expression of CDC6 was shorter than that of the patients with low CDC6 expression. Integrating the TCGA database and IHC staining, the results showed that CDC6 in ccRCC tissue was obviously up-regulated compared with adjacent normal kidney tissue. The results of Logistic regression analysis demonstrated that ccRCC patients with high expression of CDC6 are more likely to develop advanced disease than ccRCC patients with low CDC6 expression. The results of GSEA showed that the high expression of CDC6 was related to multiple signaling pathways. As for immunity, it was also related to TMB, immune checkpoint molecules, tumor microenvironment and immune infiltration. There were significantly correlations with CDC6 and immune cell infiltration levels and tumor microenvironment. The results of further results of the TCGA database showed that CDC6 was obviously related to immune checkpoint molecules and immune cells.

**Conclusions:**

Increased expression of CDC6 is a potentially prognostic factor of poor prognosis in ccRCC patients.

## Introduction

Renal cell carcinoma (RCC) is one of the most common renal malignancies. In 2020, there were an estimated 73,750 new cases and 14,830 deaths in the US (1). Recent studies demonstrated the improving incidence and mortality rates of RCC in the US (2). RCC is mainly composed of several histological subtypes—clear cell RCC (ccRCC), papillary RCC (pRCC) and chromophore RCC (chRCC) (3–5). ccRCC is the most common form of RCC and it accounts for about 70–75% of the total number of cases (6). The 5-year cancer-specific survival rate of patients with CCRCC was 68.9%, and the prognosis was worse than that of other renal cell carcinomas such as pRCC and chRCC (p <0.001) (7, 8). Localized RCC can be performed by partial or radical nephrectomy (9), ablation (10) or active monitoring (11). The diagnosis and treatment of ccRCC continue to evolve, however, at the first diagnosis, about 20 to 30% patients already have metastases (12, 13). Moreover, one-third of patients developed local recurrence or metastatic disease during long-term follow-up after resection of curable tumors (14). Nevertheless, the 5-year survival rate of metastatic ccRCC is less than 10% (15). RCC is a complex disease, and combining the biomarkers with conventional clinical pathological predictors may have important clinical significance.

**Figure 1:**
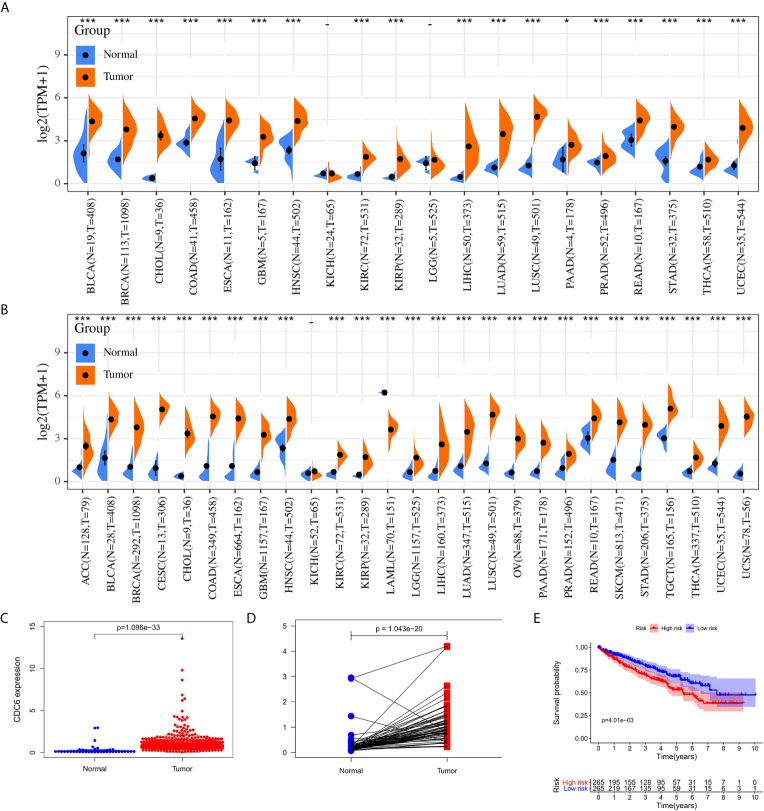
The CDC6 expression in ccRCC tissues. **(A)** The CDC6 expression in various cancers by using TCGA database. **(B)** The CDC6 expression in various cancers by using TCGA and GTEx databases; **(C)** Pairwise boxplot of the CDC6 expression between the paired normal and ccRCC tissues in TCGA dataset; **(D)** Relative expression levels of the CDC6 expression between the ccRCC and normal tissues from TCGA; **(E)** Kaplan–Meier curves for low- and high-risk groups from TCGA. *P < 0.05, ***P < 0.001.

Cell division control protein 6 (CDC6), located at chromosome 17q21.3, plays an important role in the cell cycle in the early stages of DNA replication (16–18). CDC6 is a key element of the DNA replication initiation permission system and proto-oncogene (19). Many research has shown that CDC6 has the characteristics of oncogenes, and it also plays a significant role in assessing tumor grade and predicting prognosis (19). Previous studies have shown that the down-regulation of CDC6 may inhibit E‐cadherin and metastasis of cervical cancer (20). Changes in CDC6 gene expression have been reported in many species of cancers (**Figures 1A, B**), such as gastric cancer (21, 22), pancreatic cancer (23), prostate cancer (24) and so on, related to cancer cell proliferation, metastasis, invasiveness and drug resistance. However, the role of CDC6 in ccRCC has not been described well. Therefore we analyzed the expression of CDC6 in IHC in clinical specimens of ccRCC and its relationship with clinicopathologic features and clinical outcomes in this article.

## Materials and Methods

### Data Acquisition

We acquired the expression data and related clinical information for ccRCC from The Cancer Genome Atlas (TCGA) (http://gdc.cancer.gov) datasets. All data were standardized by log2 transformation and all results were analyzed by R and GraphPad Prism 7 software. We selected |log2 fold change (FC)| ≥1 and adjusted P-value <0.05 as statistically significant genes.

### Patients and Clinical Database

We conducted a retrospective study and recruited 118 ccRCC patients who underwent radical resection surgery of ccRCC in Affiliated Hospital of Nantong University between 2015 and 2017. All patients have paraffin-embedded tissue blocks that could be used for IHC staining and outcome data. Patients were selected according to the following criteria, including 1) confirmed of histopathological diagnosis, 2) no adjuvant anti-cancer treatment after surgery, and 3) review of TNM classification. The clinicopathological data of each patient were obtained from the patient’s hospitalization records. The research medical ethics committees of the two hospitals have obtained ethics approval, and all patients participating in this study provided informed consent. All statistical tests were bilateral tests, P <0.05 was regarded statistically significant.

### Immunohistochemistry

The tissue microarray was constructed as described above. The primary anti-CDC6 antibody (diluted 1:1,000; ab109315; Abcam) was used for IHC staining. Two independent pathologists who did not know the clinical pathological data and the clinical results of each patient evaluated the staining intensity of the specimen. The specimens were deparaffinized, hydrated and blocked, and then added into the primary anti-CDC6 goat polyclonal antibody (diluted 1:1,000) and incubated overnight at 4°C. The scores were evaluation positive cells score. The positive cells score: negative: 0–5%; low: 6–25%; medium: 26–50%; high: >50%.

### Screening of CDC6 Expression and Functional and Pathway Enrichment in ccRCC

By using the Limma software package (http://www.bioconductor.org/packages/release/bioc/html/limma.html), the DEGs between tumor tissue and adjacent non-tumor kidney tissue was studied, and CDC6 expression in different clinical stages of cancer was also compared. In addition, the KEGG pathway enrichment analysis is an encyclopedia of genes and genomes through GSEA (25).

### Associations Between CDC6 Expression and MSI, TMB or Neoantigen

In order to evaluate the regulations between CDC6 expression and microsatellite instability (MSI), tumor mutational burden (TMB) or neoantigens, we applied MISA (Microsatellite, a widely-used marker system in plant genetics and forensics.) to identify all autosomal microsatellite segment composed of more than five bp in length, and introduced the specific methods as mentioned before (26, 27). Moreover, we carried out the tumor mutation burden based on the number of somatic non-synonymous mutations (NSM), and compared the sequence information between ccRCC tissues and their blood samples (28). Meanwhile, we performed seq2HLA, version 2.2 without changing the default settings to obtain the 4-digit number of differential tumors of TCGA. Then the pvac-seq was performed to produce a specific new antigen on the samples (29).

### Connection Analysis of CDC6 in Tumor Microenvironment and Immune Infiltration

To study the relationship between CDC6 gene and immune infiltration, we carried out correlation analysis with the purity-adjusted Spearman to study the correlation between six immune cell infiltration and CDC6. Besides, the ESTIMATE algorithm was implemented to estimate the three aspects consisting of the stromal, estimate and immune scores by applying the normalized expression matrix (30). P-value less than 0.001 was considered as statistically significance.

CIBERSORT is an important deconvolution algorithm used to predict the proportion of multiple cell types in multiple gene expression profiles according to reports (31). The cellular composition of the entire tissue can be estimated based on standardized data of gene expression, revealing a wealth of specific cell types (32). By evaluating each gene expression level in two aspects consisting of immune checkpoint molecules and immune cells, the gene composition in each cell was found in this study.

### Statistical Analysis

SPSS Statistics 20 was used for statistical analysis. Chi-square test was used to analyze the Categorical data. We analyzed digital data by Student’s t-test. By log-rank test compared to the subgroup OS curve calculated by Kaplan–Meier method. Univariate and multivariate Cox proportional hazards models were used to assess HR and 95% CI. All statistical tests were bilateral tests, P <0.05 was regarded statistically significant.

## Results

### Overexpressed CDC6 in ccRCC Tissues Compared With Adjacent Normal Kidney Tissues

**Figure 2:**
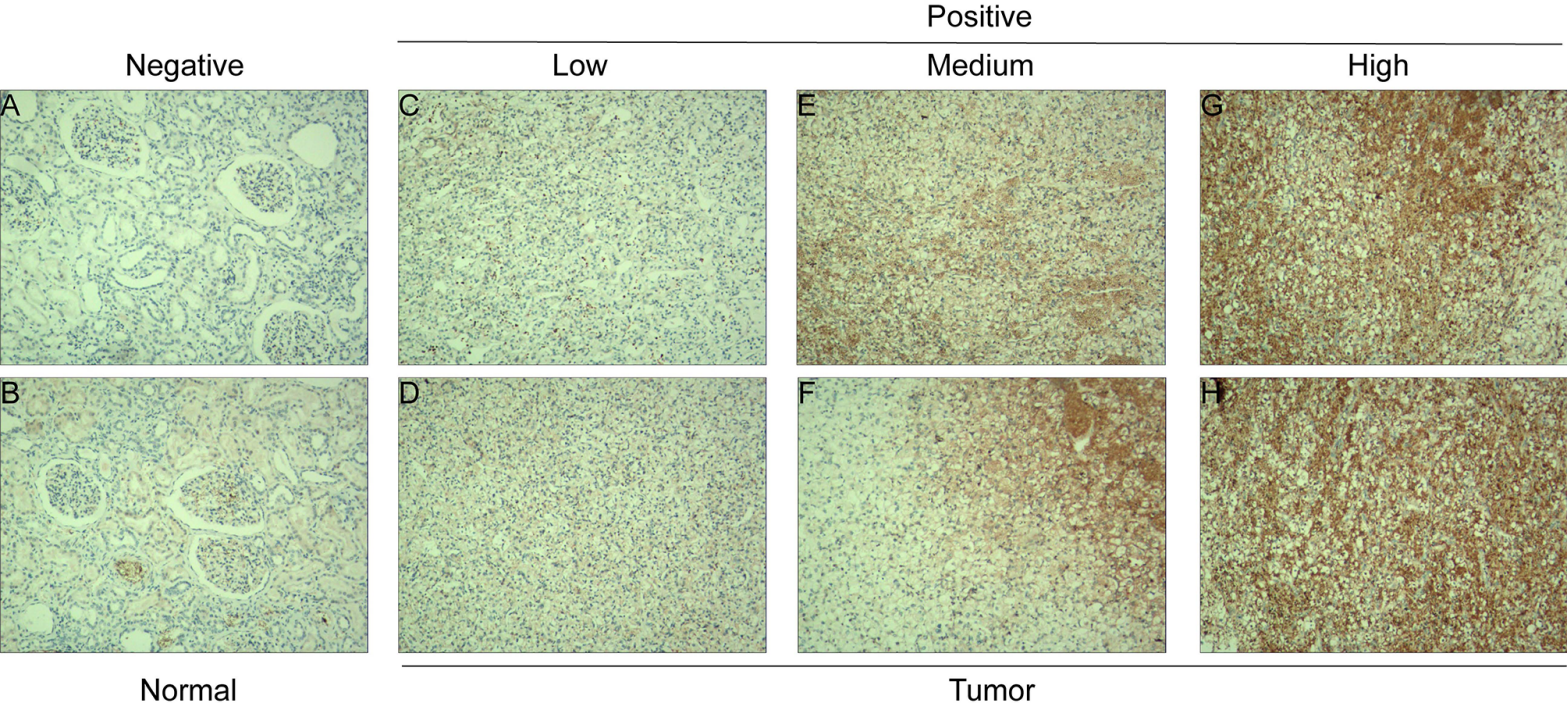
CDC6 expression in ccRCC tissues and adjacent normal renal tissues. IHC staining showed low CDC6 expression in normal renal tissues **(A, B)** and low **(C, D)**, medium **(E, F)** and high **(G, H)** ccRCC tissues.

The mRNA expression level of CDC6 was investigated to identify the differential expression pattern between tumor tissues and normal tissues from TCGA. CDC6 expression of in tumor tissues was higher than that in normal tissues (P <0.001, **Figure 1A**). Then, after analyzing the expression of CDC6 in the TCGA and GTEx data sets, the same results can be found(P <0.001, **Figure 1B**). Based on the results of ccRCC patients from the TCGA database, the expression of CDC6 was obviously increased compared with normal tissues (P <0.001, **Figure 1C**). The Pairwise boxplot also showed that most tumor samples had high expression of CDC6 (P <0.001, **Figure 1D**). Furthermore, ccRCC patients were divided into low- and high-risk subgroups according to the median expression of CDC6. The Kaplan–Meier curve indicated that the OS of the patients in the low CDC6 group was longer than that of the patients in the high CDC6 group (P <0.05; **Figure 1E**). The results of IHC staining showed that among the 118 cases(including 114 tumor cases and four normal cases), 56 cases had high expression of CDC6 levels, 58 cases had low CDC6 expression levels and four cases had negative results(all the negative results are from the normal cases) (**Figures 2A–H**). In summary, CDC6 in ccRCC tissue is significantly up-regulated compared with adjacent normal kidney tissue.

### Correlations of CDC6 Expression With Clinical Parameters in 118 ccRCC Tissues

In order to research the connection between CDC6 expression and clinicopathological parameters of ccRCC, we examined the results of IHC staining and the homologous clinical data in 118 ccRCC tissues. The clinical characteristics are shown in **Table 1**. The results showed that CDC6 expression was closely related to age (P = 0.012), tumor size (P = 0.011), T stage (P = 0.041) and Fuhrman grade (P = 0.008).

**Table 1:** Associations between CDC6 expression and clinicopathological characteristics in 118 ccRCC tissues.

Clinicopathological Variables	N	%	CDC6 Expression		P Value
			Low	High	
**Gender**					0.627
Male	66	55.9	36	30	
Female	52	44.1	26	26	
**Age, years**					**0.012**
Mean ± SD	58.8 ± 12.0		58.4 ± 12.0	62.0 ± 12.2	
Range, median	33–88.58		33–88.56	42–79.63	
**Tumor size, cm**					**0.011**
Mean ± SD	4.5 ± 1.9		4.5 ± 1.8	4.6 ± 2.0	
Range	2.0–10.0		2.0–8.0	2.0–10.0	
**T stage**					**0.041**
T1a	56	47.5	35	21	
T1b	52	44.1	23	29	
T2a	9	7.6	4	5	
T2b	1	0.8	0	1	
**Fuhrman grade**					**0.008**
1	20	15.3	16	4	
2	88	73.7	42	46	
3	8	7.6	4	4	
4	2	3.4	0	2	
**ECOG**					0.108
0	110	93.2	60	50	
≥1	8	6.8	2	6	

ECOG, Eastern Cooperative Oncology Group. The meaning of the bold values is P < 0.05 and regarded statistically significant.

### Association With CDC6 Expression and Clinicopathologic Characteristics in TCGA

**Figure 3:**
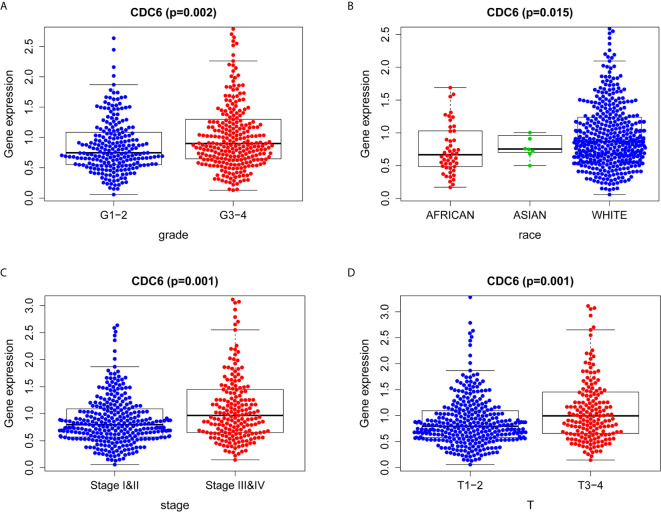
Association between CDC6 expression and clinicopathologic characteristics. **(A)** Grade; **(B)** Race; **(C)** Stage; **(D)** T stage.

Logistic regression analysis was used to explore the relationship between CDC6 expression and clinicopathological characteristics in ccRCC patients. A significant association was between high expression of CDC6 and grade (p = 0.002, **Figure 3A**), race (p = 0.015, **Figure 3B**), stage (p = 0.001, **Figure 3C**) and T stage (p = 0.001, **Figure 3D**). Therefore, ccRCC patients with high expression of CDC6 are more likely to develop advanced disease than ccRCC patients with low CDC6 expression.

### Overexpressed CDC6 in ccRCC Tissues in TCGA Database

Gene expression data and corresponding clinical information were acquired from the TCGA database. It was found that CDC6 was up-regulated in all data sets, and the results were overviewed in **Figure S1**. The results indicated that CDC6 expression in ccRCC tissue was closely associated with individual cancer stage (**Figure S1A**) and patients’ race (**Figure S1B**), patients’ gender (**Figure S1C**), patients’ age (**Figure S1D**), tumor grade (**Figure S1E**), KIRC subtypes (**Figure S1F**) and lymph node metastasis status (**Figure S1G**).

### CDC6 Could Be Regarded as an Independent Prognostic Factor

**Figure 4:**
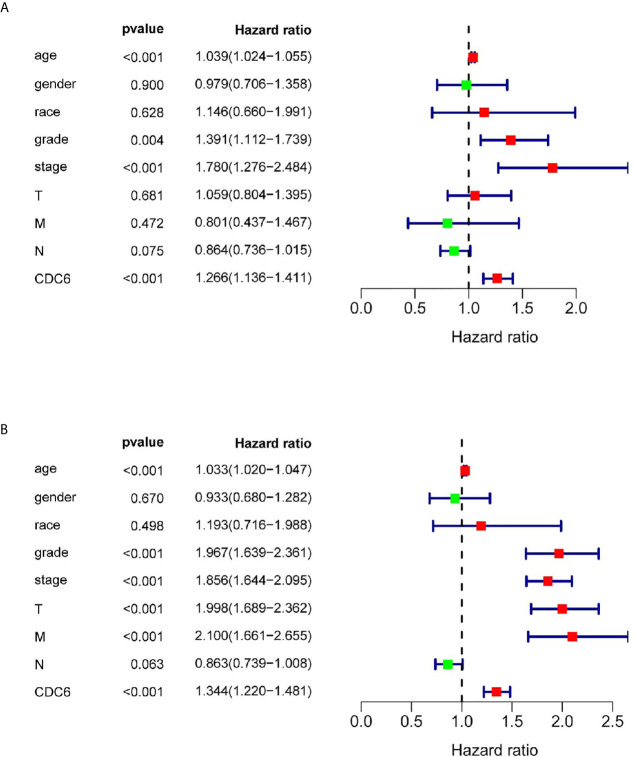
CDC6 could be regarded as an independent prognostic factor and established nomogram. **(A, B)** Univariate and multivariate Cox regression analysis of clinicopathologic variables and CDC6 in patients with ccRCC in TCGA database.

Univariate and multivariate Cox regression analysis was shown on the data from the TCGA dataset to study whether CDC6 expression is an independent factor related to OS (**Table 2**). In the univariate Cox analysis, age (HR = 1.039, p <0.001), grade (HR = 1.391, p <0.001), stage (HR = 1.780, p <0.001) and CDC6 expression (HR = 1.266, p <0.001) were independent factors related to OS in ccRCC patients (**Figure 4A**). Multivariate Cox regression analysis showed that CDC6 expression was found to be an independent risk factor for the prognosis of ccRCC patients (HR = 1.344, p <0.001; **Figure 4B**). In addition, age (HR = 1.033, P <0.001), grade (HR = 1.967, P <0.001), stage (HR = 1.856, P <0.001), T stage (HR = 1.998, P <0.001), metastasis (HR= 2.100, p <0.001) were also confirmed as an independent risk factor for OS. In summary, the above results indicated that CDC6 expression might be an independent predictor of ccRCC prognosis.

**Table 2:** Associations with overall survival and clinicopathologic characteristics in TCGA patients using univariate and multivariate Cox analysis.

Clinical characteristics	Univariate analysis		P Value	Multivariate analysis		P Value
	HR	95% CI		HR	95% CI	
Age	1.033	1.020–1.047	**0.000**	1.039	1.024–1.055	**0.000**
Gender	0.933	0.680–1.282	0.670	0.980	0.706–1.358	0.900
Race	1.193	0.716–1.988	0.498	1.146	0.660–1.991	0.628
grade	1.967	1.639–2.361	**0.000**	1.391	1.112–1.739	**0.004**
Stage	1.856	1.644–2.095	**0.000**	1.780	1.276–2.484	**0.001**
T	1.998	1.689–2.362	**0.000**	1.059	0.804–1.395	0.681
M	2.100	1.661–2.655	**0.000**	0.801	0.437–1.467	0.472
N	0.863	0.739–1.008	0.063	0.864	0.736–1.015	0.075
CDC6	1.344	1.220–1.481	**0.000**	1.266	1.136–1.411	**0.000**

The meaning of the bold values is P < 0.05 and regarded statistically significant.

### GSEA Identified CDC6-Related Signaling Pathways

To evaluate how CDC6 is involved in the pathogenesis of ccRCC, we conducted a GSEA study on the signaling pathways related to CDC6. GSEA was performed between high- and low- CDC6 expression datasets. According to the NES and FDR q-value (FDR <0.05), seven pathways showing significant differential enrichment in the high-CDC6 expression phenotype were determined and selected, including Cell cycle, Chemokine signaling pathway, Cytosolic DNA sensing pathway, JAK_STAT signaling pathway, Nod like receptor signaling pathway, P53 signaling pathway, Toll like receptor signaling pathway (**Table 3**). The results could help understand the pathogenesis mechanism underlying ccRCC.

**Table 3:** Gene sets enriched in phenotype high.

MSigDB collection	Gene set name	NES	NOM p-val	FDR q-val
c2.cp.kegg.v7.1.symbols.gmt	CELL_CYCLE	2.543	0.000	0.000
	CHEMOKINE_SIGNALING_PATHWAY	2.067	0.006	0.008
	CYTOSOLIC_DNA_SENSING_PATHWAY	2.197	0.000	0.002
	JAK_STAT_SIGNALING_PATHWAY	2.126	0.000	0.005
	NOD_LIKE_RECEPTOR_SIGNALING_PATHWAY	2.206	0.000	0.002
	P53_SIGNALING_PATHWAY	2.378	0.000	0.001
	TOLL_LIKE_RECEPTOR_SIGNALING_PATHWAY	2.299	0.000	0.001

### Associations Between CDC6 and PPI, MSI, TMB, Neoantigen in ccRCC

**Figure 5:**
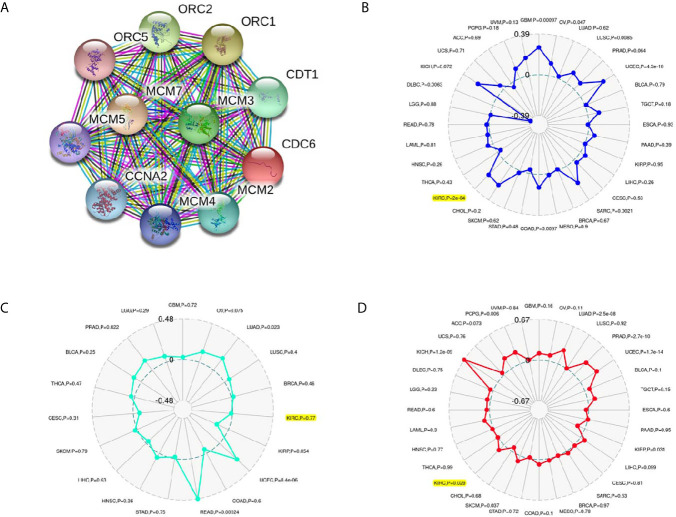
Associations between CDC6 and PPI, MSI, Neoantigen, TMB in ccRCC. **(A)** Associations between CDC6 and MSI; **(B)** Associations between CDC6 and Neoantigen; **(C)** Associations between CDC6 and TMB; **(D)** PPI network.

The PPI network showed that ten genes (ORC1, ORC2, ORC5, CDT1, MCM2, MCM3, MCM4, MCM5, MCM7 and CCNA2) were associated with CDC6 expression (**Figure 5A**). Moreover, we investigated whether CDC6 is related to MSI, neoantigen or TMB according to the ccRCC samples in the TCGA database. Our results showed that CDC6 was connected with MSI (P = 0.0002) and TMB (P = 0.029), but not connected with neoantigens (P = 0.77) (**Figures 5B–D**).

### Associations Between CDC6 and the Immune Infiltrations and Tumor Microenvironment in ccRCC

**Figure 6:**
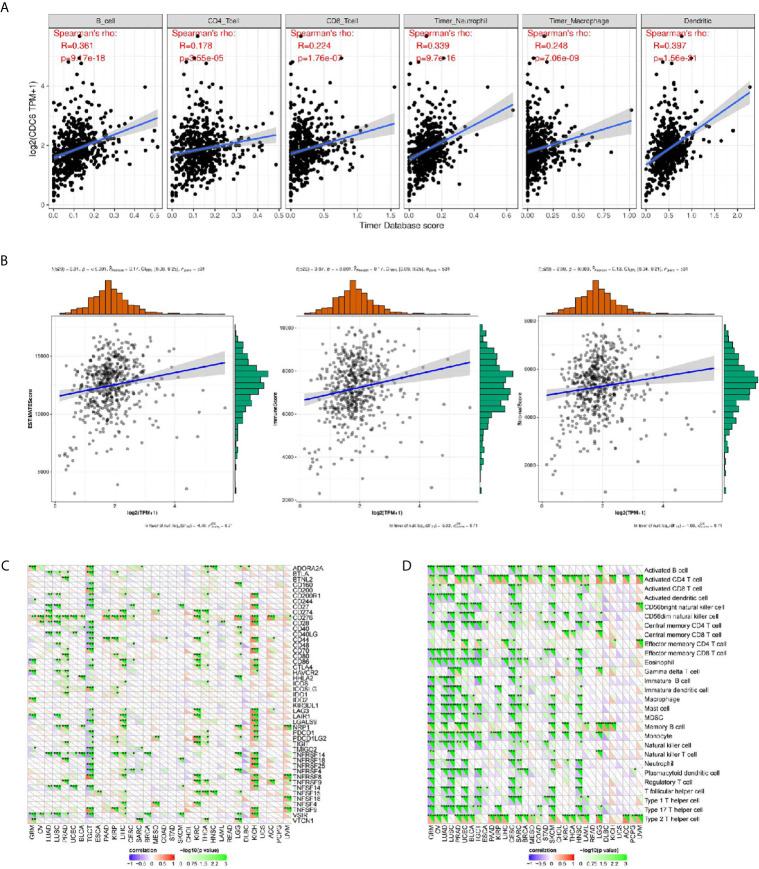
Associations between CDC6 and the immune infiltrations, tumor microenvironment, immune checkpoint molecules and immune cells. **(A)** Associations between CDC6 and immune infiltrations; **(B)** Associations between CDC6 and immune microenvironment; **(C)** Associations between CDC6 and immune checkpoint molecules; **(D)** Associations between CDC6 and immune cells. *P < 0.05, **P < 0.01, ***P < 0.001.

Through online analysis TIMER, the correlation were conducted between CDC6 and six immune cell infiltration levels, it was found that CDC6 was significantly related with the immune infiltrations consisting of B cell infiltration, CD4+ T cell infiltration, CD8+ T cell infiltration, neutrophil infiltration, macrophage infiltration, and dendritic cell infiltration (P <0.01, **Figure 6A**). In addition, it showed that CDC6 has significant relationships with immune cells, stromal cells, and both of them (**Figure 6B**).

### Associations Between CDC6 and Immune Checkpoint Molecules and Immune Cells

In order to further study the association between CDC6 and the immune microenvironment of ccRCC tissues from the TCGA database, we analyzed more accurately and found that CDC6 was obviously related to the immune checkpoint molecules, such as CD274, CD276, CD444, CD80 etc. in ccRCC (**Figure 6C**). Moreover, we investigated the immune pathway between CDC6 and immune cells in ccRCC and showed that CDC6 is significantly connected with immune cells, including Active CD4 T cell, Central memory CD8 T cell, Memory B cell etc. (**Figure 6D**).

## Discussion

CDC6 plays an important role in the activation and maintenance of checkpoint mechanisms in the cell cycle by acting as a regulator at the early stages of DNA replication (16–18). It has been reported that the changes in CDC6 gene expression in many species of cancers, such as gastric cancer (21, 22), pancreatic cancer (23), prostate cancer (24), are related to the proliferation, metastasis, invasiveness and drug resistance of cancer cells. However, few studies have concentrated on the correlation between the CDC6 gene and the prognostic prediction of ccRCC.

Hence, our study reported the correlation between the high expression of CDC6 and the high risk of death in ccRCC patients for the first time. The high expression of CDC6 is positively correlated with Fuhrman grade and tumor T stage, which strongly indicates that CDC6 plays a vital role in the occurrence and development of ccRCC.

In this study, an analysis of RNA sequence data in ccRCC from TCGA was carried out systematically. Compared with CDC6 expression level in normal renal tissues, we found that CDC6 expression in tumor tissues was elevated. According to the median expression of CDC6 in all patients, we divided patients with ccRCC from TCGA into low- and high-expression groups. The results demonstrated that the OS in the high-expression group was shorter than that in low-expression group.

The results of IHC staining indicated that CDC6 in ccRCC tissue was significantly up-regulated compared with adjacent normal kidney tissue. Furthermore, the results of the corresponding clinical data showed that CDC6 expression is closely related to age, tumor size, T stage and Fuhrman grade. Logistic regression analysis showed that there was a significant association between high expression of CDC6 and grade, race, stage and T stage. According to TCGA and ICGC databases, we found CDC6 expressed highly in tumor tissues compared with adjacent normal tissues. It was found that CDC6 was up-regulated in all data sets. The results showed that the CDC6 expression in ccRCC tissue is closely related to individual cancer stages, patients’ race, patients’ gender, patients’ age, tumor grades, KIRC subtypes, and lymph node metastasis status. Moreover, univariate and multivariate Cox regression analysis demonstrated that CDC6 gene has the potential to be a predictor of the prognosis of ccRCC patients.

In order to study the possible signaling pathways and mechanisms of CDC6, we conducted GSEA analysis and found a total of seven related pathways were identified. These pathways showed significant differential enrichment in the high-CDC6 expression phenotype, including Cell cycle, Chemokine signaling pathway, Cytosolic DNA sensing pathway, JAK_STAT signaling pathway, Nod like receptor signaling pathway, P53 signaling pathway, Toll like receptor signaling pathway.

As for immunity, CDC6 was significantly related to MSI and TMB. It was found that CDC6 was related to the immune infiltrations including B cell infiltration, CD4+ T cell infiltration, CD8+ T cell infiltration, neutrophil infiltration, macrophage infiltration, and dendritic cell infiltration. Furthermore, it showed that CDC6 was closely related to immune cells and stromal cells. This study determined that CDC6 expression could be a prognostic factor for ccRCC patients.

In addition, CDC6 was significantly related to immune checkpoint molecules in ccRCC such as CD274, CD276, CD444, and CD80. Besides, we studied the immune pathways between CDC6 and immune cells in ccRCC, and it indicated that CDC6 was strongly connected with related immune cells, including active CD4 T cell, Central memory CD8 T cell, Memory B cell etc.

There are still a few limitations that ought to be attached importance to. First and foremost, clinical data was limited, because the data from TCGA was retrospective. Second, in TCGA, the sample size of normal kidney tissues was relatively small, which may lead to our conclusion bias. Last but not least, we do not yet know the likely mechanism of CDC6. For example, is it endogenous or exogenous, or both? We need a larger sample size and enough clinical data to correct our results in future studies.

In conclusion, our study determined that CDC6 expression was a potential significant poor prognostic indicator in ccRCC patients. In addition, Cell cycle, Chemokine signaling pathway, Cytosolic DNA sensing pathway, JAK_STAT signaling pathway, Nod like receptor signaling pathway, P53 signaling pathway, Toll like receptor signaling pathway may be the main regulation of CDC6 way. Moreover, CDC6 is closely associated with immunity and it could be regarded as an independent prognostic factor of ccRCC. We need subsequent basic researches to confirm our findings *in vivo* and *in vitro*. Further research may verify whether CDC6 can be developed as a new therapeutic target.

## Data Availability Statement

The original contributions presented in the study are included in the article/**Supplementary Material**. Further inquiries can be directed to the corresponding authors.

## Ethics Statement

The studies involving human participants were reviewed and approved by Ethics Committee of Affiliated Hospital of Nantong University. The patients/participants provided their written informed consent to participate in this study. Written informed consent was obtained from the individual(s) for the publication of any potentially identifiable images or data included in this article.

## Author Contributions

YY planned, designed and performed the experiments and wrote the manuscript. YW analyzed the data and involved in manuscript preparation. DW and BH supervised the research and checked the manuscript. All authors contributed to the article and approved the submitted version.

## Funding

This study was funded by grants from National Natural Science Foundation of China (81802560, 81672526). All these study sponsors have no roles in the study design, in the collection, analysis, and interpretation of data.

## Conflict of Interest

The authors declare that the research was conducted in the absence of any commercial or financial relationships that could be construed as a potential conflict of interest.

## Supplementary Material

Supplementary Figure 1
**(A–G)** CDC6 expression in ccRCC tissues is strongly related with individual cancer stages, patients’race, patients’gender, patients’age, tumor grade, KIRC subtypes and nodal metastasis status. **P < 0.01, ***P < 0.001.
