# Corrigendum: Increased CDC6 Expression Associates With Poor Prognosis in Patients With Clear Cell Renal Cell Carcinoma

**DOI:** 10.3389/fonc.2021.731942

**Published:** 2021-07-21

**Authors:** Yicong Yao, Yi Wang, Denglong Wu, Baoying Hu

**Affiliations:** ^1^ Department of Urology, Tongji Hospital, School of Medicine, Tongji University, Shanghai, China; ^2^ School of Medicine, Tongji University, Shanghai, China; ^3^ Department of Urology, Affiliated Hospital of Nantong University, Nantong, China; ^4^ Department of Immunology, Medical College, Nantong University, Nantong, China

**Keywords:** CDC6, prognosis, clear cell renal cell carcinoma, survival, pathway

In the published article, there was an error in affiliation 2 and affiliation 4. Instead of affiliation 2 Shanghai Tongji Hospital, Tongji University School of Medicine, Shanghai, China, it should be Department of Urology, Tongji Hospital, School of Medicine, Tongji University, Shanghai, China. Instead of affiliation 4 Department of Immunology, Medical College, Nantong University, Shanghai, China, it should be Department of Immunology, Medical College, Nantong University, Nantong, China.

Furthermore, we exchanged the order of Affiliations 1 and 2. Therefore, the new Affiliation 1 is 1 Department of Urology, Tongji Hospital, School of Medicine, Tongji University, Shanghai, China and the new Affiliation 2 is 2 School of Medicine, Tongji University, Shanghai, China.

The authors apologize for this error and state that this does not change the scientific conclusions of the article in any way. The original article has been updated.

