# Supplementary figures and images for: Increased CDC6 Expression Associates With Poor Prognosis in Patients With Clear Cell Renal Cell Carcinoma
Source: Front Oncol. 2021 May 24;11:666418. doi: 10.3389/fonc.2021.666418 (PMC8202290; doi:10.3389/fonc.2021.666418)

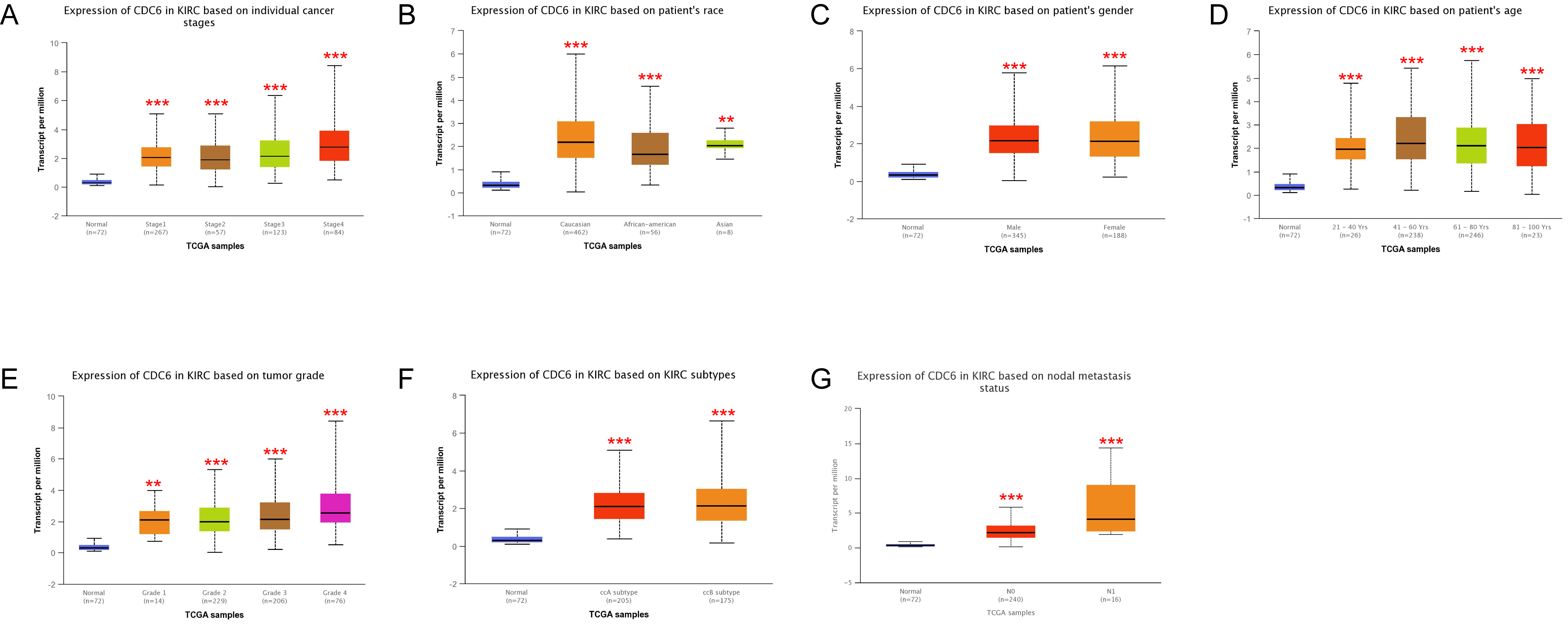

Supplement: Supplementary Figure 1 — (A–G) CDC6 expression in ccRCC tissues is strongly related with individual cancer stages, patients’race, patients’gender, patients’age, tumor grade, KIRC subtypes and nodal metastasis status. **P < 0.01, ***P < 0.001. [file Image_1.tif]
